# Effectiveness of capacity building interventions relevant to public health practice: a systematic review

**DOI:** 10.1186/s12889-018-5591-6

**Published:** 2018-06-01

**Authors:** Kara DeCorby-Watson, Gloria Mensah, Kim Bergeron, Samiya Abdi, Benjamin Rempel, Heather Manson

**Affiliations:** 1 Public Health Ontario, 480 University Avenue, Suite 300, Toronto, ON M5G 1V2 Canada; 2 School of Rehabilitation Therapy, Queen’s University, 31 George St., Kingston, ON K7L 3N6 Canada; 3 School of Public Health and Health Systems, University of Waterloo, 200 University Avenue West, Waterloo, ON N2L 3G1 Canada; 4 Dalla Lana School of Public Health, University of Toronto, 155 College Street, 6th floor, Toronto, ON M5T 3M7 Canada

**Keywords:** MeSH (7) capacity building, Professional competence/st [standards], Public health, Total quality management/og [Organization & Administration], Health education/st [standards], Health promotion/st [standards], Global Health

## Abstract

**Background:**

This systematic review assessed the effectiveness of capacity building interventions relevant to public health practice. The aim is to inform and improve capacity building interventions.

**Methods:**

Four strategies were used: 1) electronic database searching; 2) reference lists of included papers; 3) key informant consultation; and 4) grey literature searching. Inclusion (e.g., published in English) and exclusion criteria (e.g., non-English language papers published earlier than 2005) are outlined with included papers focusing on capacity building, learning plans, or professional development plans within public health and related settings, such as non-governmental organizations, government, or community-based organizations relating to public health or healthcare. Outcomes of interest included changes in knowledge, skill or confidence (self-efficacy), changes in practice (application or intent), and perceived support or supportive environments, with outcomes reported at the individual, organizational or systems level(s). Quality assessment of all included papers was completed.

**Results:**

Fourteen papers were included in this review. These papers reported on six intervention types: 1) internet-based instruction, 2) training and workshops, 3) technical assistance, 4) education using self-directed learning, 5) communities of practice, and 6) multi-strategy interventions. The available literature showed improvements in one or more capacity-building outcomes of interest, mainly in terms of individual-level outcomes. The available literature was moderate in quality and showed a range of methodological issues.

**Conclusions:**

There is evidence to inform capacity building programming and how interventions can be selected to optimize impact. Organizations should carefully consider methods for analysis of capacity building interventions offered; specifically, through which mechanisms, to whom, and for which purpose. Capacity-building interventions can enhance knowledge, skill, self-efficacy (including confidence), changes in practice or policies, behaviour change, application, and system-level capacity. However in applying available evidence, organizations should consider the outcomes of highest priority, selecting intervention(s) effective for the outcome(s) of interest. Examples are given for selecting intervention(s) to match priorities and context, knowing effectiveness evidence is only one consideration in decision making. Future evaluations should: extend beyond the individual level, assess outcomes at organizational and systems levels, include objective measures of effect, assess baseline conditions, and evaluate features most critical to the success of interventions.

**Electronic supplementary material:**

The online version of this article (10.1186/s12889-018-5591-6) contains supplementary material, which is available to authorized users.

## Background

Fast-paced globalization of health challenges the capacity of the public health field to not only adopt evidence, but also to adapt it locally to keep pace with public health events [1, 2]. Globalization raises questions about public health capacity to respond to communicable and non-communicable diseases, inequalities and their impact on health and disease, global health governance, climate change, economic events, and moving from siloed to integrated approaches across sectors [3]. Public health is also faced with responding to new technologies, the dissemination of which could deteriorate social and environmental context by widening inequalities and contributing to unhealthy lifestyles [4].

Despite demands placed on public health to meet new and future challenges, skill deficits in the public health workforce are evident and include insufficient preparation via education and training for the jobs performed, and an overreliance on experience and on-the-job trial and error [5]. Self assessments of public health competency by agency workers and their supervisors consistently show gaps between mastery and what is needed for effective practice [6]. These gaps are documented in areas corresponding to key competencies, including the use of evidence in decision making (e.g., economic evaluation, communicating with policymakers, evaluation designs, and adapting interventions) [7].

Given the deficits reported, there is a need for capacity building. Capacity building is defined by the World Health Organization (WHO) as “the development of knowledge, skills, commitment, structures, systems and leadership to enable effective health promotion...[with] actions to improve health at three levels: the advancement of knowledge and skills among practitioners; the expansion of support and infrastructure for health promotion in organizations, and; the development of cohesiveness and partnerships for health in communities” [8]. Engaging in these actions is called a capacity building intervention [9]. The aim of this type of intervention is to improve the practice of public health practitioners and the infrastructure of public health organizations by enhancing and sustaining individual and organizational capacity to address local health issues [10]. It requires future planning, and systems that can meet surge capacity, with continuous training, beyond the education required to achieve public health qualifications [11]. Capacity building interventions can take a variety of forms including providing technical assistance, in-depth consultations, virtual and in-person training sessions, online learning options, guidance materials in the form of knowledge products, and skills-based courses [12] among others such as coaching and mentoring [13–15].

Efforts to foster public health capacity have included the development of core competencies, credentialing, accreditation, and a number of policy recommendations, which include the systematic assessment and development of the public health workforce. [5, 16–20] Developing capacity building interventions has been a role taken on by various agencies at different levels including WHO and the World Federation of Public Health Associations (WFPHA) at an international level, the Public Health Agency of Canada (PHAC) and Centers for Disease Control and Prevention (CDC) as national-level examples, and international and national non-governmental organizations (NGOs) (e.g. World Health Organization, Planetary Health Alliance, UN Foundation), health promotion and prevention resource centres and community-based organizations at a local level.

There appears to be limited research on the effectiveness of capacity building interventions related to individual and organizational capacity building in public health. One systematic review was found that explored community-based interventions; however, this review was limited to community-based interventions and studies published in academic journals, with included papers not quality appraised [21]. To our knowledge, a more comprehensive and broader assessment of the effectiveness of capacity building interventions in public health related to individual and organizational capacity building has not been conducted. Evidence on the effectiveness of these strategies would ideally be available to factor into choices about which strategies to apply in different public health contexts.

The purpose of this systematic review is to assess the effectiveness of capacity building interventions relevant to public health practice. The aim is to inform and improve capacity building interventions.

## Methods

A framework for completing the review was constructed in advance and included the following steps: 1) search strategy, 2) determining inclusion and exclusion criteria, 3) screening papers, 4) methodological quality assessment of primary studies or reviews, followed by 5) data extraction and 6) synthesis of results [22]. A protocol is not published for this review.

### Search strategy

Four strategies were used: 1) electronic database searching; 2) reference lists of included papers; 3) key informant consultation; and 4) grey literature searching. A systematic electronic search was conducted by Public Health Ontario (PHO) Library Services on September 29, 2015 and updated on September 29, 2016 in four databases: 1) Ovid MEDLINE, 2) Embase, 3) CINAHL Plus with Full Text, and 4) PsycINFO. The search aimed to locate articles on the impact of capacity building in public health and general healthcare and included “Capacity building” [MeSH] as well as keywords “capacity building”, “prevention capacity”, “health promotion or public health”, “build or increase capacity” and “learning plan”. See Additional file 1 for the full search strategy. We searched reference lists of all included articles and conducted key informant consultation to identify additional references that might have been missed. Key informants included PHO’s Health Promotion Capacity Building team members and managers of Ontario Health Promotion Resource Centres. A grey literature search was conducted on November 10, 2016 and included grey literature repositories, custom web search engines, and a general web search (see Additional file 2). Appropriate databases for the search were determined in consultation with health science librarians based on the subject coverage required. The published database search strategy covered much of the indexed literature for Web of Science (WOS) and Google Scholar (GS). Beyond health databases, PsycINFO further broadens the evidence base to include psychology and behavioural sciences. The grey literature strategy extended the search beyond academic publishing, significantly expanding its scope.

### Inclusion and exclusion criteria

Articles and reports were included if they were published in English over the last 11 years, were about capacity building interventions such as learning plans, or professional development plans within public health or related settings (e.g. non-governmental organizations, government, or community-based organizations relating to public health or healthcare), offered summary-level evidence (guidelines), synthesis-level research or primary studies that included evaluations of capacity building interventions, and included outcome data related to effectiveness which could be organized and analyzed according to intervention type (e.g. training and workshops, technical assistance). Outcomes of interest included changes in knowledge, skill or confidence (self-efficacy), changes in practice (application or intent), and perceived support or supportive environments, with outcomes reported at the individual, organizational or systems level(s). Outcomes measures were determined by the authors prior to the review work, and reflected relevant measures for the health promotion and public health system. Commentaries were eligible for inclusion if they reported data on outcomes.

Exclusion criteria included non-English language papers published earlier than 2005, articles focusing on curriculum development in academic settings (university research centres and departments), acute care facilities, hospitals, long-term care centres, agricultural agencies, afterschool agencies, community development and community capacity building interventions, articles that did not provide outcome data, and interventions in low resource and/or low-to-middle income countries related to obtaining credits for continuing professional education or medical education, or capacity building .

### Screening and selection of studies

#### Electronic database

Titles and abstracts of all identified articles in the original 2015 search were screened by two review authors (KD and KB), who independently screened 20% of the search results for relevance and had an agreement score greater than 80%. The remaining 80% of results were split in half and independently screened (KD and KB). Two team members (KD and GM) independently screened the full set of the updated search conducted in 2016. At full-text relevance screening, two authors (KD and GM) independently screened 20% of all full-texts, and the remaining 80% was screened by one author (GM). Any discrepancies were resolved by discussion until consensus was reached, and occasionally a third reviewer from the team, or the full research team, was consulted. The reference lists of all relevant articles were searched to identify additional articles. Any potentially-relevant additional papers were retrieved in full document versions and screened for inclusion.

#### Grey literature and key informant consultation

Titles and abstracts of all grey literature search results were screened by one author (KD). All sources identified through key informant consultation were screened for relevance by one author (GM). Full-text assessment was independently carried out by two authors (KD and GM). Consensus was reached on all discrepancies via discussion between the two authors.

#### Quality assessment

Methodological quality was independently assessed by two authors (KD and GM) using criteria appropriate to the design of the included papers. Specifically, the Health Evidence Quality Assessment Tool for systematic reviews and meta-analyses was used for systematic review and/or meta-analysis articles [23]. Articles that used a qualitative methodology were appraised using the criteria for appraising qualitative research studies developed by Walsh and Downe [24]. Quantitative studies were assessed using the Quality Assessment Tool for Pre and Post Intervention Designs by Brown et al., which is adapted from Estabrooks et al. [25, 26] and studies applying mixed methodology were assessed with the Mixed Methods Appraisal Tool (MMAT) [27]. All disagreements on individual quality ratings were discussed until consensus was reached. All of the tools used allowed the researchers to rate an article’s quality as strong, moderate or weak by identifying risk of bias. Regardless of research design or tool used, all articles rated weak in methodological quality were excluded from this review, with the exception of one included article, which was the only paper that reported outcomes at the systems level [28]. Where this paper is mentioned, results are placed in context with a statement about quality. Quality appraisal results for included papers are shown in Appendix C. Ratings displayed in each table are the final ratings agreed on by both reviewers. Each quality appraisal table indicates the range of quality scoring that resulted in weak, moderate, and strong ratings.

#### Data extraction

A data extraction table was drafted to meet the purpose of the project, and refined by discussion among the research team. The form was then piloted and discussed by two authors (GM, KD). The resulting discussion generated a guide for data extraction with instructions for completing each item, to achieve consistency. Data extraction was then completed by one author (GM) and reviewed by a second author (KD) to ensure that appropriate data were extracted from all included papers. Authors were not contacted to validate data published in the included papers. Information extracted from each paper included: author and year of publication, purpose or objective, type of intervention, population and setting, providers, study design, context, theories and frameworks cited, theories and frameworks used to develop interventions, measurement tool, outcomes, findings/results, implications for practice, and study limitations. Findings included a range of outcome measures determined to be relevant to the health promotion and public health capacity building field and are outlined with their methods of measurement in Table 1.

**Table 1:** Range of outcomes and methods of measurement for included papers

Outcomes	Measurement strategies applied in included articles
Knowledge	• Meta-analysis (including effect size or standardized mean difference) • Interviews (telephone, focus groups, key informant) • Review of documents (relevant documents, planning and implementation documents) • Questionnaire (online or hardcopy) • Written reflections
Understanding	• Questionnaire
Skill	• Meta-analysis (effect size, standardized mean difference) • Interviews (telephone, focus groups, key informant) • Review of documents (relevant documents, planning and implementation documents) • Questionnaire (online or hardcopy)
Leadership (self-efficacy)	• Interviews (telephone)
Confidence (self-efficacy)	• Interviews (telephone, key informant) • Review of documents (relevant documents) • Questionnaire (online or hardcopy) • Pre-post tests • Evaluation tool
Changes in practice and policies	• Interviews (telephone) • Questionnaire (online or hardcopy)
Behaviour change	• Questionnaire (derived from CARE measure and Cognitive Therapy Scale)
Application	• Interviews (focus groups) • Online survey • Written reflections
Organizational support	• Interviews (telephone)
Perception of system level capacity	• Trauma System Readiness Tool (TSRT) • Online surveys

#### Data analysis

We conducted a systematic review that focused on the effectiveness of all included papers relevant to the impact of interventions for capacity building. For the purpose of this paper, a systematic review is defined as an evidence synthesis that adheres to guidelines on the conduct of a review [29]. Our review referred to the process for systematic review set out by the Cochrane Health Promotion and Public Health Field [30]. For quantitative studies, the primary outcome measure was the mean difference or percentage change in knowledge, skill or confidence, and qualitative reporting of change at the individual, organizational, or systems level(s). However, a quantitative analysis of results (meta-analysis) was not carried out due to differences in measurement tools and scales, diverse outcome measures, and the mix of quantitative and qualitative data. Both qualitative and quantitative data were synthesized together, for a single narrative description of impact taking into account both the quantitative and qualitative results, for each intervention type. Given the objective of summarizing effectiveness evidence for strategies, we synthesized results by intervention type and describe them here according to each strategy reported in the currently available literature.

## Results

**Fig. 1:**
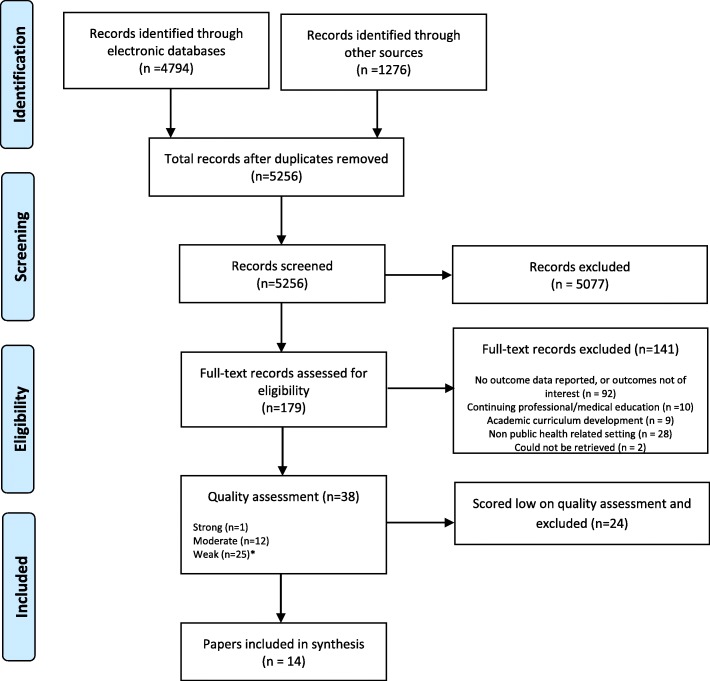
PRISMA flowchart diagram of included papers. The PRIMSA flowchart details our search, screening and inclusion decisions made during the review process. *Inclusion of one low quality paper as it was the only one focused on systems level change

A total of 38 papers were assessed as relevant and then appraised for methodological quality, with 24 scoring low in quality and being excluded from the synthesis due to lack of quality. The remaining 14 papers were included. For appraising quality, an appropriate tool was selected for each of the research designs present. Tools were selected for systematic review quality appraisal (including meta-analysis), quantitative studies, qualitative studies, and mixed methods designs. Please see the PRISMA flowchart (Fig. 1) for an overview of the process.

After removal of all weak-quality papers except the one included that featured outcomes at the systems level, 14 papers were included in the review. These 14 papers reported outcomes related to the impact of a variety of types of capacity building interventions, mainly focused on individual-level interventions as opposed to organization-level or system-level interventions. Characteristics of included papers are shown in detail in Additional file 3: Tables S1 and Additional file 4: Table S2.

Individual-level interventions were reported most often and included internet-based instruction, self-directed educational interventions or curriculum, workshops with and without mentoring support, core competency and specialized skill training modules, in-person training using case scenarios and individual reflection, and technical assistance. In several cases, multi-component strategies were evaluated and included various combinations of interventions like technical assistance, mentoring, communities of practice, online discussion groups, case analysis, small group work, and role play. Only one paper reported system-level interventions, with the interventions reported being workforce development, changes to screening practice, revisions to practice and policy, and dissemination of evidence-based practices [28]. These intervention types are further defined in the sections that follow.

The capacity building interventions reported in the current literature were provided for a range of audiences including health professionals (physicians, nurses, residents, social workers, occupational therapists), staff at community-based and national organizations, community health workers, public health staff/community health staff, some education sector staff (combined along with health/community staff), and national health service staff. Providers for these interventions included expert consultants, evaluators (manager or leader), clients, multidisciplinary teams, health professionals (e.g. psychologists, diabetologist, specialist general practitioners), resource centre staff, public health staff, researchers or research centres, academic institution representatives and community health centre staff, and in two papers, providers were unspecified.

The types of research designs used to evaluate capacity building interventions include: systematic review (two meta-analyses), mixed methods, qualitative, and quantitative methods, represented by simple before-after evaluations. The range of outcomes and measurement methods for the included studies are shown in Table 1. They are presented in the order of most available evidence in the literature to least available evidence.

In terms of quality of the available literature (Appendix C), review-level papers (meta-analyses) were well-done with no serious flaws identified after quality appraisal. Quantitative evaluations were moderately well-done, with none rated high in quality. Quantitative papers’ flaws included a general lack of controls, failure to measure at multiple time points (simple pre-post), failure to use reliable or valid tools to measure, use of self-report rather than objective measurement of outcomes, and lack of rationale for drop outs from their projects.

All included papers that were qualitative in nature rated moderate in quality, and none rated high in quality. Qualitative papers’ flaws typically were that they did not explain rationale for selection of a particular method, lacked an audit trail to support understanding what was done to obtain results, lacked reflection/explanation about the possible impact of the researcher or person doing the intervention/data collection on the data itself and on the participants/intervention, and failed to consistently address ethical concerns. Mixed methods papers featured a combination of the flaws identified in both the quantitative and qualitative literature separately.

As part of data extraction, we recorded whether authors cited a particular theory or framework related to their research. Notably, only four of 14 papers cited a particular framework, with no overlap in theories/frameworks cited across the four papers [31–34]. However, all of the four that clearly referenced a framework also applied it in their work. We assessed “application” by requiring that, in order to have applied it, authors must have described how it was applied in their work as evidence that there was use of the theory or framework that was instrumental to the intervention, beyond simple conceptual use in thinking about the activity of capacity building. In each of the four papers that specified particular theories or frameworks, these papers all applied the theory or framework in designing the intervention (e.g., strategies used, content, delivery methods) [31–34]. Theories and frameworks cited and applied appear in Additional file 4: Table S2 Characteristics of included papers [insert hyperlink to Additional file 4: Table S2 here].

The following sections report outcomes organized by each of the six intervention types noted in the currently available literature: 1) internet-based instruction, 2) training and workshops, 3) technical assistance, 4) education using self-directed learning (SDL), 5) communities of practice (CoP), and 6) multi-strategy interventions. Each intervention type reports a description of the intervention results for that intervention type by each outcome.

### Internet-based instruction

A well-done meta-analysis by Cook et al., incorporated 201 individual studies that assessed 214 separate interventions [35]. The review evaluated whether internet-based instruction enhanced knowledge and skill for capacity building, comparing internet-based interventions to no intervention, and also comparing internet to non-internet based interventions. The interventions were aimed at the individual level, for health professional learners (residents, doctors, nurses, dentist, pharmacists, and others). This review’s inclusion criteria for interventions was deliberately broad and included any computer-assisted instruction using the internet as the mode of delivery [35]. Cook et al. reported two outcomes of interest: knowledge and skills, and found that internet-based instruction greatly improved knowledge and skills compared to no intervention, but not compared to other forms of instruction [35]. Findings follow in detail for each outcome with a description of factors that influence the success of internet-based instruction on each outcome.

#### Knowledge

Compared to no intervention, internet-based instruction showed a large effect on knowledge (ES 1.00, 95% CI 0.90–1.10, *p* < 0.001) [35]. In terms of features of internet-based instruction that worked best, there was evidence of a larger effect for those that included a discussion component vs. no discussion (*p* = 0.002), and for longer courses (*p* = 0.03) [35]. Level of interactivity, provision of practice exercises, and repetition did not appear to have a significant impact.

Compared to other forms of instruction (face to face, modules on paper, self-study modules, videoconferences), internet-based instruction offered a statistically significant benefit but the effect was quite small (ES 0.12, 95% CI 0.003–0.24, *p* < 0.05) [35]. Thus overall, internet-based instruction had a large impact compared to no intervention but did not offer a meaningful advantage compared to other forms of intervention.

#### Skills

Similar to the impact of internet-based instruction on knowledge, compared to no intervention, there was a large effect on skills as an outcome (ES 0.85, CI 0.49–1.20, *p* < 0.001). Practice exercises seemed to enhance the effect of internet-based instruction on skills, while interactivity, repetition and discussion did not influence outcomes.

Also aligned with results related to knowledge, internet-based instruction showed no benefit to skill development when compared to other forms of intervention (ES 0.09, 95% CI -0.26 – 0.44, *p* = 0.61) [35].

### Education using self-directed learning (SDL)

Although curricula were not included and analyzed as part of this review, we were interested in education programs that used self-directed learning (SDL). A single, well-done meta-analysis reviewed SDL for capacity building [36]. The interventions included in the review were any self-directed methodology delivered to health professionals and meeting three specific criteria of Knowles’ determinants of self-directedness: teachers must have acted as facilitators as opposed to content sources, learners being involved in selecting learning resources and strategies, and learnings involved in self-assessment of their own learning outcomes [36]. Subgroup and sensitivity analyses showed some facilitators of SDL impact that for learners who are more involved in learning resource selection, and for those who are more advanced in learning, make greater improvements. Interactivity of learning modules did not make a significant difference in SDL impact, nor did length of SDL or time between end of intervention and evaluation of outcomes [36].

#### Knowledge and skill

Murad’s meta-analysis showed a moderate, significant increase in knowledge (SMD 0.45, 95% CI 0.23–0.67) using self-directed learning, compared to traditional teaching methods and no significant skill increases for SDL strategies over traditional teaching methods [36]. The review did not assess whether knowledge increases demonstrated via SDL were sustained beyond the post-intervention evaluation measurements.

### Training and workshops

For the purpose of analysis, training and workshops were grouped together since their definitions in the literature didn’t allow for distinctions between interventions labelled ‘training’ and what was considered ‘workshops’.

Seven papers reported training and workshop outcomes that included: knowledge, understanding, skill, confidence (self-efficacy), changes in practice and policy, behaviour change, and application of knowledge [32–34, 37–40]. Overall, training and workshops achieved gains in knowledge, skill, confidence, change in practices and policies (single study evidence), and behaviour (single study evidence). We did not find evidence to support the enhancement of understanding. A summary of findings for each outcome follows.

#### Knowledge

Four papers assessed knowledge after training and workshops [32, 34, 37, 39]. Of these, three reported qualitative and quantitative knowledge increases, in terms of increasing knowledge in a current content area and gaining new knowledge [32, 34, 37]. The paper reporting quantitative knowledge gains did not compare data pre and post, or by intervention and control groups; as such, it did not offer a comparison with confidence interval or *p* value. However, knowledge ‘scores’ were considered high if 8 or greater on an eleven-point Likert scale [32]. Conflicting with the three papers showing knowledge increases was a pre-post comparison that found no significant increase in knowledge scores (*p* = 0.986) [39]. Both quantitative papers reported conflicting findings for very similar interventions; both described a series of five training sessions aimed at health service staff (in Ireland and Canada), and featured a mentoring/facilitation component as follow up support to the training sessions provided [32, 39]. Both quantitative and qualitative data were gathered via self-report. Qualitative findings were consistently positive and show value in training and workshops for enhancing knowledge [32, 34, 37].

#### Understanding

A single paper reported increased understanding after a second of two training sessions, with the impact sustained after 5 months post-intervention; however, there were significant differences between groups at baseline [40]. These differences limit the strength and generalizability of the findings. This methodological limitation combined with the lack of other review-level or single study evidence limits our ability to draw a conclusion about training and workshops to enhance understanding [40]. Since authors did not define knowledge an understanding, we did not consolidate knowledge and understanding where reported separately as two distinct outcomes.

#### Skill

Three papers reported consistently positive, quantitative skill gains for training and workshops [32, 34, 39]. While Keogh did not use comparison data (pre-post or intervention-control) [32], both Matthews and Swanson report quantitative skill enhancement in a number of specific areas [32, 34, 39]. These include Matthews’ skill gains for writing a research question (*p* = 0.003), critically reading research literature (*p* = 0.047), developing a research study (*p* = 0.008), developing a data collection tool (*p* = 0.033); [39] and use of positive communication/behaviour change techniques (*p* = 0.0001) [34]. Swanson also measured use of negative behaviour change techniques using a number of specific behaviours based on a widely-accepted taxonomy, finding use of negative techniques decreased from baseline to post-workshop (*p* = 0.0001) [34].

#### Confidence/self-efficacy

All three papers that evaluated this outcome reported positive gains in confidence/self-efficacy, with all three reporting qualitative findings and two also reporting quantitative findings, with findings from each of the two methods validating each other. Descriptive (qualitative) findings showed increased confidence reported by participants as:

- increased confidence and comfort with their content area (sexual health) [32],
- increased confidence and motivation to include (sexual health) promotion activities within their roles [32],
- confidence in their ability to use skills, intentions related to applying learning and changing their approach [33], and
- greater confidence to use skills and feelings of empowerment and motivation [34].

Ruiz reported a 23% increase in confidence [33], with Keogh indicating confidence scores were within a range designated as high [32]. While quantitative findings were also positive, in line with qualitative results, the two papers reporting quantitative findings did not assess whether differences in findings before and after the training/workshop were significant, so it is difficult to assess whether differences were due to the training/workshop activity [32, 33]. Regardless, consistently positive qualitative findings alongside these quantitative findings are an indication that capacity building training and workshops can enhance confidence.

#### Changes in practice and policy

A single study by Keogh reported on practice and policy change with both quantitative and qualitative outcomes with quantitative change assessed as percentage change [32]. Both its quantitative and qualitative findings showed positive practices and policy change. In quantitative outcomes, the study did not provide the data (*p* values, confidence intervals) to assess whether differences observed were significant; however, engagement across all activities showed increases. For the purpose of highlighting potentially-meaningful gains, we note that four areas showed large gains of 30% or greater, in terms of the proportion of participants active in capacity-building practices at the individual, organizational, and inter-organizational levels after the training compared to prior [32]. These were: (individual) attending training or education on sexual health promotion (increased by 30%); (organizational) awareness raising of sexual health promotion needs within the organization (increased by 41%); (organizational) provision of formal sexual health education to staff (increased by 31%); (inter-organizational) networking with other individuals and organizations (increased by 40%) [32]. Qualitative findings corroborate this, indicating actions at the organizational and inter-organizational levels that were enhanced after the training including: conducting workshops and training for staff, use of facilitation skills, staff discussion on sexual health topics, policy and guideline development, and inter-organizational activities such as journal publications, workshops done for other organizations, training for volunteers, formal and information networking and conducting research [32]. Though a single study reported these findings, results suggest capacity building training and workshops can enhance practices and policies related to their target content.

#### Behaviour change

A single study reported behaviour change as an outcome evaluated qualitatively via questionnaire (with both scaled and open-ended feedback) [34]. Participants reported already having implemented changes in their own behaviours [34]. While behaviour change was measured less often than other outcomes, capacity building training and workshops appear to be a potential intervention to impact behaviour in a positive way.

#### Application

Application of knowledge from capacity building training and workshops was evaluated by two studies that reported consistent positive results. Qualitative findings showed community health workers immediately applied knowledge from training by educating family, community, and other community health workers [37]. Additionally, a post-training questionnaire (no pre-test) showed what proportion of participants used evidence-based public health (EBPH) content from the training in the past months (time interval for post-questionnaire not indicated) [38]. Of all staff who attended, a low but potentially meaningful proportion indicated that, every month since the training, they engaged in EBPH behaviours such as searching scientific literature (35.7%), referring to EBPH readings (22.4%), and using EBPH materials for grant applications (3.1%), as well as for program planning (26.5%), modification (24.5%), and evaluation (23.5%) [38]. The project did not assess the extent to which the roles of participating local health department staff required or overlapped with the specific EBPH behaviours measured. Although the number of months over which the behaviours were repeated is unknown, this project establishes the potential of training and workshops to result in their content being applied to some extent [38].

### Technical assistance

Two papers qualitatively evaluated technical assistance (TA), with both reporting individual-level knowledge and skill, leadership, and confidence [41, 42]. Both papers conceptualized TA as personalized support, including face to face meetings, for tobacco-control system staff in Canada, and the United States. Findings for actual changes in practice, and for organizational support, were mixed [41, 42]. Following are more detailed descriptions of the findings from both papers, related to those five outcomes.

#### Knowledge and skill

In both settings, TA clients reported knowledge gains relevant to their content area, including the development of new skills such as enhanced communication and presentation skills, overcoming barriers at council meetings, and media and communication [41, 42].

#### Confidence

Both Kegler and Lambracki reported increased confidence in one’s own work, with Lambracki also reporting commitment to take on new challenges and overcome barriers [41, 42]. For example, Kegler reports that increased confidence came from knowing there was direction for the work and that work was not simply reactionary [41].

#### Change in practice and policy

In the area of practice and policy change, TA resulted in service improvement, foundational work for policy action, increased use of evidence, and enhanced collaboration (via development of partnerships and network building) [41]. These findings were tempered with participants’ mixed reviews on whether or not TA increased support from policy makers, with some or no increase as a result of TA that could undoubtedly impact policy change [41].

#### Organizational support

At an organizational level, TA clients reported neutral or mixed feelings about the impact of TA services on organizational support [41]. However, for both dimensions of organizational support evaluated, there were explanations for mixed or neutral findings that imply increased organizational support may not have been an expected outcome. In evaluating support from major decision makers as one dimension of overall organizational support, participants reported they either had a lot of support already (so support had not increased); their work with the TA hadn’t targeted program leaders; they were able to make the necessary decisions themselves; or support hadn’t yet been established but may have been in development [41]. Although most reported mixed or neutral feelings about support, some reported organizational support increased, which was attributed to leaders’ participation, enhanced understanding and confidence, and provision of useful information to leaders. [41] When asked about the second dimension of organizational support evaluated, keeping and obtaining funding, participants’ responses were overall negative, saying it had not contributed; however, the most common rationale was that it still remained to be seen or “not yet”. With telephone interviews occurring approximately 3 months after the TA service, it is reasonable that impact on funding decisions and overall organizational support would have been difficult to assess via this study’s timeline [41]. Kegler also evaluated leadership change and found strengthened leadership abilities [41]. Though it identified the key objective of TA as building organizational or community capacity, the second study did not evaluate the impact of TA at the organizational level, and the description of services and TA utility was mainly focused on individual clients [42].

### Communities of practice

A single paper reported outcomes for communities of practice (CoP) [31]. Although a large body of literature has reported data for communities of practice, this was the only paper located that focused on CoP for the purpose of capacity building. In this instance, the intervention targeted individual occupational therapists (OT) working with children and youth who were distributed across different practice and geographic settings [31]. A lead occupational therapist and co-facilitators led the CoP over 6 months, during which the intervention focused on relationships between researchers and practitioners, active learning strategies (reading, face to face, and online discussions), and shared learning and problem solving, with sharing of resources and new strategies. Three face to face meetings (start, middle, end) were held in a central location, with six online discussions aimed at facilitating reading, reflection, and sharing. A summary of the CoP activities are reported in more detail in the following sections.

#### Knowledge

Bazyk reported significant knowledge gains measured quantitatively via pre and post-test results, finding that CoP participation increased knowledge statements (*p* < 0.00 for all four knowledge statements evaluated) [31]. Although not a knowledge outcome, belief statements relating to staff members’ own ability to, and feasibility of, addressing mental health, and others’ awareness about mental health showed significant improvement as well (*p* < 0.00 and *p* < 0.002 across three statements), and action statements related to knowledge about their own practices (p < 0.00 for both statements) from pre to post test [31]. Knowledge gains were accompanied by self-reported changes in practices and policies, measured qualitatively via analysis of documents and final reflections, including:

- Capacity building being perceived as meaningful and enjoyable (described changes to daily practice; perception of support via networks)
- Changes in thinking because of new knowledge in mental health (“reframing”, “paradigm shift”, plans to use new knowledge)
- Experience evoking strong emotions related to practitioners’ own identities (reconnection to mental health roots was rewarding, enhanced awareness of scope of practice)
- Reported practice changes (ways of working shifted, including large changes to their own work such as undertaking a new, year-long program, and joining new initiatives) [31]

This mixed-methods study reported changes at post-test and did not report whether knowledge gains and reported practice changes were sustained [31].

### Multi-strategy interventions

Two papers evaluated multi-strategy interventions [28, 43]. For the purpose of this review, multi-component strategies were those which explicitly described the intervention as being multi-component or consisting of multiple strategies. Both papers explicitly defined their interventions as having multiple components; a training component and written materials. Other components included technical assistance; CoPs; mentoring/coaching; internship; appreciative inquiry; use of technology; meetings, and the application of a Collaborative (Connecticut Collaborative on Effective Practices for Trauma; CONCEPT), screening, trauma informed policy and practice guide revisions, and evidence based practices (EBP) dissemination [28, 43].

#### Knowledge

Knowledge improved via multi-component, capacity building interventions [28, 43]. Preskill’s mixed methods evaluation found most participants reported improved evaluation skills (e.g. developing logic models, understanding of terms and concepts, significance of findings for decision making, range of data collection methods, and evaluation process generally [43]. Similarly, Lang found that staff trauma knowledge and practice improved (Mean 0. 26, 95% CI 0.11 to 0.40, *p* < 0.01), and individual trauma knowledge and practice also improved significantly (Mean 0. 33, 95% CI 0.20 to 0.46, *p* < 0.01) [28].

#### Skill

In addition to knowledge, skills were also enhanced by multi-component interventions [43]. All participants in Preskill’s mixed methods study reported qualitative improvements, with staff asking better questions and using evaluation findings more after the intervention [43]. Some staff were also reported to design better data collection tools and communicate with and report to stakeholders more efficiently [43].

#### System readiness and capacity

Lang evaluated a system-level, multi-component intervention delivered over 2 years and aimed at generating system-level change in evidence-informed trauma care [28]. Pre-post evaluation found significant improvement in perceived readiness and capacity between years one and three. Readiness and capacity were operationalized and enhanced in a variety of ways including trauma training and education (Mean 0. 39, 95% CI -0.23 to 0.54, *p* < 0.01), trauma supervision and supports (Mean 0. 52, 95% CI 0.33 to 0.0.71, p < 0.01), access to trauma-informed services (Mean 0. 51, 95% CI 0.33 to 0.69, p < 0.01), and local agency collaboration (trauma) (Mean 0. 31, 95% CI 0.15 to 0.48, p < 0.01) [28]. There was no significant difference in local agency collaboration (general) (Mean 0. 05, 95% CI -0.06 to 0.16, non-significant) [28]. Although we rated the methods used for evaluation as weak in quality (Appendix C), this paper offers an example of a longer-term intervention with options for meaningful, system-level outcomes that can be evaluated to inform programming, beyond solely individual outcomes.

## Discussion

This systematic review assessed public health capacity building interventions for their relative impact on knowledge, skill, self-efficacy (including confidence), changes in practice or policies, behaviour change, application, organizational support, and perception of system-level capacity, to inform capacity building practices and services at multiple levels. Given the time frame over which capacity building interventions have been in place (and capacity building defined), the evaluations available in the current literature were surprisingly sparse. More consistent evaluation of the interventions would offer a firm foundation upon which capacity-building organizations can base decisions about how they deliver services.

For the evaluations reported in the current literature, methodological quality was low to moderate, without high-quality in any of the four research designs located and appraised, with the exception of the meta-analysis by Cook et al. [35]. Given half of all potentially-relevant papers could be excluded based on low quality suggests there is a need to strengthen evaluation of capacity-building interventions. Further, not only more consistent, but also better evaluations are needed. Specifically, evaluations should consider more rigorous designs moving beyond pre and post-test approaches, using methods such as multi-time point data collection, and longer-term data collection that would capture some measure of sustainability for the strategy under investigation. Evaluations should consider data collection methods beyond self-report, or validation measures to strengthen confidence in their outcomes and decrease the potential risk of bias known to be associated with self-reported measures (particularly where self-report is the only measure). Measures used in evaluation should reflect dimensions of the theory or framework underpinning the capacity building intervention [9]. Although we saw theories and frameworks applied in planning of the intervention for the included papers, they were not used instrumentally in the evaluation of those interventions. Additionally, most evaluations only measured knowledge and skill change rather than actual behaviour change, or change in practice and policy.

The range of evaluated interventions represents the scope of what is commonly referred to as capacity building in a public health context. However, the papers remain focused on capacity building at the individual level, despite acknowledgement that individual behaviour is shaped by interaction with the environment at multiple levels [44], and calls to move toward system-level action [45, 46]. Over 10 years after the 2006 WHO definition called for capacity building to happen at multiple levels, it could be expected that evaluations of capacity building would also take into account those multiple levels, and would offer some organization and system-level data. However the current literature included in this paper does not reflect this. A lack of system-level focus in the available literature also contrasted sharply with the call for system focus in addressing the new public health landscape [45, 46]. A 2016 analysis of 55 public health measurement cases showed a similar focus on individual-level outcomes, finding that system-level outcomes tended to be omitted altogether since attribution was more difficult at that level [47]. Thus, attempting evaluation using organizational and system-level measures and potential proxy measures, and referring to papers that evaluate outcomes at those levels for examples of how to evaluate, should be considered.

Each of the six interventions included in the current literature found improvements in one or more capacity-building outcomes of interest. A large, well-done meta-analysis found that internet-based capacity building enhanced both knowledge and skill, but only in comparison to no other intervention [35]. Important features for improving knowledge were the inclusion of discussion groups, while practice sessions made a greater difference to skill building outcomes [35]. Level of interactivity and repetition made no difference for either knowledge or skill enhancement [35]. Given this, internet-based interventions offer an advantage where no other interventions are feasible or accessible such as in remote geographic regions. However, where a number of different delivery systems for capacity building are available, internet-based interventions would not offer an advantage over existing interventions that may be more feasible, given the investment required to set up online learning systems and the supporting infrastructure. Where knowledge is the priority outcome to influence, discussion groups should be part of an internet-based intervention, and where skill enhancement is required, developers should incorporate practice sessions. However, the knowledge that the real impact of internet-based interventions happens in the absence of other interventions means that it is important to consider audience needs, what else they might have access to, and whether the outcome of interest at the individual level is knowledge or skill (or both).

Self-directed learning was also evaluated via meta-analysis, and enhanced knowledge but made no significant difference in skill. Self-directed learning may prove an effective strategy where knowledge alone is the outcome of interest, but where skill gains are expected, programs should consider alternate strategies. That SDL could impact knowledge in a significant way is promising since it represents a first step or initial outcome that could be built upon in a phased approach or in a series of interventions to advance learners along a continuum, potentially reaching behaviour change and practice and policy change at later stages. It also offers the advantage of requiring fewer resources than more intensive interventions like technical assistance.

Training and workshops were evaluated by seven included studies that showed enhancements across almost all outcomes including knowledge, skill, confidence, improvements in practice and policy, behaviour change, and application of knowledge [32–34, 37–40]. Only understanding was not shown to be enhanced, and this was due to significant differences between groups at baseline. Those implementing training and workshops should consider carefully the implementation of the interventions where behaviour change, improvements in practice and policy, and application of knowledge were demonstrated. Identifying the key elements of those interventions would support a move toward organization and system-level evaluation.

Technical assistance was defined consistently by two studies that evaluated its impact [41, 42]. A consistent definition not only facilitates the implementation of TA but also allows for more straightforward comparison across evaluations. TA improved knowledge, skill, practice and policy change, without conclusive results for organizational support. A short time frame for evaluation since the end of the intervention may not have been sufficient to either expect or measure organizational support change [41]. Also, participants’ comments indicate that baseline conditions may have been quite favourable, making it even less likely that meaningful additional change would have occurred [41]. It will be important to evaluate baseline conditions and consider a feasible level of expected change in future studies, while still evaluating organization-level variables.

The potential of TA to impact actual practice and policy change is meaningful in that it represents an outcome beyond the individual level. Practice and policy change was also affected by training and workshops, so the features and potential complementary nature of the two strategies could be considered where practice and policy change are goals.

Multi-component interventions were consistent in enhancing both knowledge and skill; however, there was limited evidence, and the evidence did not report on which key elements contributed most to their observed impacts. It is unknown which components of multi-strategy interventions most influenced their impact. Multi-strategy interventions should offer some assessment of which components were critical, and which combination of interventions is optimal. Those ideas were not explored in the available literature, but would contribute to awareness of which combination of strategies would work best together to impact which outcomes, and at which level(s).

Findings from this review support the effectiveness of capacity-building interventions to impact knowledge, skill, self-efficacy (including confidence), changes in practice or policies, behaviour change, application, and perceptions of system-level capacity. However, this support exists mainly at the individual level. Despite lack of evidence for capacity building impact beyond the individual level; impacts on behaviour change, practice and policy change, and application are promising.

### Review limitations and strengths

There may be other relevant documents beyond published articles and grey literature searches, which are not available in the public domain and/or there may be articles published in another language on this topic. This systematic review’s limitations relate mainly to the state of the current, available literature. The literature was searched for English language papers only. Limitations of the literature at a study level include the quality of the available literature overall and the fact that evaluation methods used were not strong, with some consistent methodological issues: the extent to which measurement tools were reliable and valid, and data consistently relying on self report. The literature is not clear about the role of public health staff in capacity building, with a lack of clarity on providers. There was consistently greater focus on describing the target audience in setting the approach, with less information about the role of capacity-building providers.

A limitation at the review level was that the sectors from which evidence was drawn were limited to those related to public health and health promotion specifically. Having reviewed the evidence from the health sectors only, our search strategy could have missed literature in related fields such as implementation science, secondary education, and knowledge utilization as examples. However, this review’s focus was on the public health context, and this review established the state of the literature nearest to that context, based on the most relevant and current papers.

Strengths of this review include the author’s collective experience working in a capacity building organization, the use of a comprehensive search strategies (e.g., four were used) and assessment of the quality of included articles.

### Implications for practice

Although data gaps exist and most evaluation rests at the individual level, the public health field must still take action. We should apply the available effectiveness evidence to programming and planning moving forward, and continue to advance the evaluation related to capacity building.

Based on current evidence about what works, capacity building programming should consider which outcomes are of highest priority. Priority setting done by a working group or other body, or determined via prioritization of audience needs could be useful in seeing which interventions could offer the best fit. High-priority outcomes could be aligned with intervention strategies that offer the best chance of achieving those outcomes. An assessment of learning objectives that aligned outcomes with the most effective intervention(s) and consideration of context may also be useful. For example, if delivering interventions in remote regions or where there aren’t currently options available, internet-based interventions may work well and be the most feasible. In contrast, where a host of interventions are available, other factors such as outcome goals matched with the evidence on what works may be weighted more heavily. The evidence also provides direction to optimize delivery of interventions; for example, when applying SDL, involving learners in their own learning resource selection results in greater improvements, as well as targeting those who are more advanced in learning. Thus, SDL may be particularly appropriate, and feasible, for capacity building beyond introductory level material.

Challenges related to budgets and funding commitments, available resources, and other organizational considerations will undoubtedly influence how capacity building is done and evaluated. Interventions themselves can require a high volume of resources, particularly where the intervention involves web development, moderation for discussion groups, or technical assistance as an example of a more personalized (and in-person) approach to capacity building. Where outcomes of interest can be achieved equally well via a less intense option, such as training and workshops, the latter option may be more viable.

Opportunities for long-term planning lie in combining the practical considerations that accompany the effectiveness evidence in selecting the most relevant, appropriate interventions, and then determining whether the desired impact is achieved and sustainable, to contribute to what is known about how capacity building interventions work. Given certain outcomes are reported mainly at the individual level, once an intervention is selected, those evaluating capacity building interventions should work to further advance the evidence. The current literature can help by offering strategies for conceptualizing measures beyond the individual level. Organization and systems-level measurement strategies such as those used to operationalize Lang’s system capacity and readiness offer an example [28]. Working toward a more complete body of evidence showcasing outcomes on multiple levels would support more informed decision making around capacity-building interventions and how best to deliver them.

## Conclusions

Ongoing training and multi-level strategies are needed in developing and delivering programs, and to adapt and measure effectiveness of their interventions [48]. This measurement imperative must extend to evaluating the capacity building strategies implemented to help public health reach its goals in a changing landscape.

While a range of capacity building interventions have been set in place and have become standard, there is less evaluation of which intervention work best in a public health or health promotion context. It is unclear based on currently-available literature how capacity building interventions are selected and applied, and where they are used in the absence of evaluation data to show they work. Organizations should carefully consider methods for analysis of capacity building interventions being offered, through which mechanisms they work best, for whom, and for what purpose.

Regardless of the need for enhanced evaluation and consideration of effectiveness evidence in implementation, there is clear advantage to particular interventions. Capacity building does enhance outcomes related to the work of public health. In future it will be important to assess how these can be influenced at different levels beyond that of the individual and considering how strategies work for entire organizations and systems that operate in different contexts.

## Additional files

Additional file 1: Appendix A and B. Search strategy examples. Full Medline search strategy and a general web search for grey literature. (DOCX 52 kb)

Additional file 2: Appendix C. Results of quality assessment of the included 14 papers with final ratings. (DOCX 26 kb)

Additional file 3: **Table S1.** (Characteristics of included papers) to be linked approximately here (DOCX 37 kb)

Additional file 4: **Table S2.** (Intervention types and outcomes) to be linked approximately here (DOCX 51 kb)

## Abbreviations

CBO: Community-based organization
CDC: Centers for Disease Control and Prevention
CHW: Community health worker
CoP: Community of practice
IUHPE: International Union for Health Promotion and Education
MMAT: Mixed Methods Appraisal Tool
NGO: Non-governmental organizations
OT: Occupational Therapists
PHAC: Public Health Agency of Canada
SDL: Self-directed learning
WFPHA: World Federation of Public Health Associations
WHO: World Health Organization
