# Supplementary material for: Effectiveness of capacity building interventions relevant to public health practice: a systematic review
Source: BMC Public Health. 2018 Jun 1;18:684. doi: 10.1186/s12889-018-5591-6 (PMC5984748; doi:10.1186/s12889-018-5591-6)
Supplement: Supplementary file 1 — Appendix A and B. Search strategy examples. Full Medline search strategy and a general web search for grey literature. (DOCX 52 kb) [file 12889_2018_5591_MOESM1_ESM.docx]

**Appendix A**

A systematic electronic search was conducted September 29, 2015 in Ovid MEDLINE (1946 to Present), Embase (1974 to 2015 Week 39), CINAHL Plus with Full Text, and PsycINFO (2002 to September Week 3 2015). An update to the electronic search was run September 29, 2016.

| **#** | **Searches** | **Results** |
| --- | --- | --- |
| **1** | Capacity Building/ or ("capacity building" or "prevention capacity" or (system* adj2 capacit*) or (("health promotion" or "public health") adj5 capacit*) or ((build* or increas* or develop* or enhanc* or strengthen*) adj5 (capacit* or skill* or abilit* or workforce)) or "learning plan*").ti,kw,kf. | 8744 |
| **2** | Diffusion of Innovation/ or Knowledge Management/ or Models, Educational/ or Models, Nursing/ or Models, Organizational/ or Models, Psychological/ or Models, Theoretical/ or Systems Theory/ or (theor* or framework* or construct? or model* or concept* or heuristic* or lens or paradigm* or principle? or pre-engagement or phase? or stage? or "innovation support").ti,kw,kf. or (theor* or framework* or construct? or model* or concept* or heuristic* or lens or paradigm* or principle? or pre-engagement or phase? or stage? or "innovation support").ab. /freq=2 | 2224244 |
| **3** | Competency-Based Education/ or Computer-Assisted Instruction / or Consultants/ or Education, Distance/ or Education, Professional/ or Education/ or Educational Technology/ or Evidence-Based Medicine/ or Evidence-Based Nursing/ or Evidence-Based Practice/ or exp Education, Continuing/ or Government Programs/ or exp Health Personnel/education or Health Planning Technical Assistance/ or Inservice Training/ or Learning/ or Mentors/ or Planning Techniques/ or Preceptorship/ or Program Development/ or Program Evaluation/ or Staff Development/ or Teaching/ or ("best practice*" or "change agent*" or "learning plan*" or "promising practice*" or (evidence adj3 (base* or inform*)) or approach* or assist* or consultant* or educat* or effectiv* or elearn* or implement* or initiative* or instruct* or intervention* or learn* or mentor* or preceptor* or program* or strategies or strategy or support* or teach* or tool or tools or train* or webinar* or workshop* or "technical assistance").ti,kw,kf. or ("best practice*" or "change agent*" or "learning plan*" or "promising practice*" or (evidence adj3 (base* or inform*)) or approach* or assist* or consultant* or educat* or effectiv* or elearn* or implement* or initiative* or instruct* or intervention* or learn* or mentor* or preceptor* or program* or strategies or strategy or support* or teach* or tool or tools or train* or webinar* or workshop* or "technical assistance").ab. /freq=2 | 2964774 |
| **4** | Health Occupations/ or Health Personnel/ or manpower.fs. or Personnel Delegation/ or Personnel Management/ or Personnel Selection/ or Personnel Turnover/ or Professional Autonomy/ or Professional Competence/ or Professional Practice/ or ("human resource*" or employee* or employer* or manpower or personnel or practitioner* or professional* or provider* or staff or worker* or workforce).ti,kw,kf. | 331406 |
| **5** | Community Health Planning/ or Health Care Reform/ or Health Planning Technical Assistance/ or Health Planning/ or Health Systems Agencies/ or Health Systems Plans/ or Organizational Case Studies/ or Organizational Culture/ or Organizational Innovation/ or Organizational Objectives/ or Regional Health Planning/ or Systems Analysis/ or (system or systems or systemic or "health care" or "health administration" or (health adj3 (plan* or reform*)) or organization*).ti,kw,kf. | 845591 |
| **6** | Health Promotion/ or Public Health Administration/ or Public Health Practice/ or Public Health/ or ("public health" or "health promot*" or "health unit*" or "health authorit*" or "health department*" or "community health" or "population health").ti,kw,kf. | 183438 |
| **7** | 1 and (2 or 3) and (4 or 5 or 6) | 1887 |
| **8** | limit 7 to english language | 1839 |
| **9** | limit 8 to last 10 years | 1484 |
| **10** | (exp Africa/ or exp Caribbean Region/ or exp Central America/ or exp Latin America/ or exp South America/ or exp Asia/ or Mexico/ or Developing Countries/) not (North America/ or exp Canada/ or exp United States/ or exp Australia/ or New Zealand/ or exp Europe/ or exp Developed Countries/) | 845509 |
| **11** | 9 not 10 | 1238 |
| **12** | remove duplicates from 11 | 1186 |

**Appendix B: Grey literature search terms**

**Google**  [www.google.com](http://www.google.com)

| **Search strategy** | **Date searched** | **# results retrieved** | **# results reviewed** | **# results selected** |
| --- | --- | --- | --- | --- |
| "public health" OR "health promotion” capacity OR skill OR ability OR workforce  build OR increase OR develop OR enhance OR strengthen | Nov 10,2016 | 154,000,000 | 50 | 7 |
| "public health" OR "health promotion” capacity OR skill OR ability OR workforce  build OR increase OR develop OR enhance OR strengthen | Nov 10,2016 | 154,000,000 | 50 | 1E |
| "public health" OR "health promotion” capacity building change-agent OR consultant OR workshop OR webinar | Nov 10,2016 | 2,800,000 | 50 | 2E |
| "public health" OR "health promotion” capacity building theory OR framework OR model OR concept OR heuristic OR paradigm OR principle OR stage OR lens | Nov 10,2016 | 3,720,000 | 50 | 4 |
| "public health" OR "health promotion” capacity building learning OR teaching OR instruction OR education OR training OR mentor OR preceptor | Nov 10,2016 | 4,140,000 | 50 | 3 |
| "public health" OR "health promotion” capacity building human-resources OR employee OR employer OR manpower OR health-personnel OR health practitioner OR health-professional OR health-provider OR staff OR workforce OR health-system | Nov 10,2016 | 39,800,000 | 50 | 0 |
| "prevention capacity" OR "health promotion capacity" OR "public health capacity" | Nov 10,2016 | 126,000 | 50 | 0 |
