# Supplementary material for: Effectiveness of capacity building interventions relevant to public health practice: a systematic review
Source: BMC Public Health. 2018 Jun 1;18:684. doi: 10.1186/s12889-018-5591-6 (PMC5984748; doi:10.1186/s12889-018-5591-6)
Supplement: Supplementary file 2 — Appendix C. Results of quality assessment of the included 14 papers with final ratings. (DOCX 26 kb) [file 12889_2018_5591_MOESM2_ESM.docx]

# Appendix C

**Health Evidence Quality Assessment Tool for Systematic Reviews and Meta-analyses**

| **Criterion** | **Cook et al 2008** | **Murad et al 2010** |
| --- | --- | --- |
| **Quantitative reviews (10 points)** | | |
| 1. Did the authors have a clearly focused question [population, intervention (strategy), and outcomes(s)]? (1 point) | Yes | Yes |
| 1. Were appropriate inclusion criteria used to select primary studies? (1 point) | Yes | Yes |
| 1. Did the authors describe a search strategy that was comprehensive? (1 point) | No | No |
| 1. Did search strategy cover an adequate number of years? (1 point) | Yes | Yes |
| 1. Did the authors describe the level of evidence in the primary studies included in the review? (1 point) | Yes | Yes |
| 1. Did the review assess the methodological quality of the primary studies? (1 point) | Yes | No |
| 1. Are the results of the review transparent? (1 point) | Yes | No |
| 1. Was it appropriate to combine the findings of results across studies? (1 point) | Yes | Yes |
| 1. Were appropriate methods used for combining or comparing results across studies? (1 point) | Yes | Yes |
| 1. Do the data support the author's interpretation? (1 point) | Yes | Yes |
| **Total Score** | **9** | **7** |
| **Rank** | **Strong** | **Moderate** |

**Note: Total score calculation (total number of points obtained); Highest possible score is 10. Reviews with a score of 8 or higher were rated strong, a score between 5-7 as moderate, and a score of 4 or less as weak.**

**Criteria for appraising qualitative research studies (Walsh & Downe, 2006)**

| **Criterion** | **Brady & Keogh, 2015** | **Kegler & Redmon, 2015** | **Lambraki et al 2015** | **Preskill & Boyle, 2008**** |
| --- | --- | --- | --- | --- |
| **Scope and purpose (2 points)** | | | | |
| 1. Clear statement of, and rationale for, research question/aim/purposes (1 point) | Yes | Yes | Yes | Yes |
| 1. Study thoroughly contextualized by existing literature (1 point) | No | No | Yes | No |
| **Design (2 points)** | | | | |
| 1. Method/design apparent, and consistent with research intent (1 point) | Yes | No | No | No |
| 1. Data collection strategy apparent and appropriate (1 point) | Yes | Yes | Yes | Yes |
| **Sampling strategy (1 point)** | | | | |
| 1. Sample and sampling method appropriate (1 point) | Yes | Yes | Yes | Yes |
| **Analysis (1 point)** | | | | |
| 1. Analytic approach appropriate (1 point) | Yes | Yes | Yes | Yes |
| **Interpretation (3 points)** | | | | |
| 1. Context described and taken account of in interpretation (1 point) | Yes | Yes | Yes | No |
| 1. Clear audit trail given (1 point) | No | Yes | No | No |
| 1. Data used to support interpretation? (1 point) | No | No | No | Yes |
| **Reflexivity ( 1 point)** | | | | |
| 1. Researcher reflexivity demonstrated (1 point) | No | Yes | No | No |
| **Ethical dimensions ( 1 point)** | | | | |
| 1. Demonstration of sensitivity to ethical concerns (1 point) | Yes | Yes | Yes | Yes |
| **Relevance and transferability ( 1 point)** | | | | |
| 1. Relevance and transferability evident (1 point) | Yes | No | Yes | Yes |
| **Total Score** | **8** | **8** | **8** | **7** |
| **Rank** | **Moderate** | **Moderate** | **Moderate** | **Moderate** |

**Note: Total score calculation (total number of points obtained). Highest possible score is 12. Specific prompts were provided for each criterion to assist in rating. Articles and papers with a score of 10 or higher were rated strong, a score between 6-9 as moderate, and a score of 5 or less as weak. **Study authors identify methodology as mix methods; article reports findings from a larger study presenting only qualitative data.**

**Quality Assessment Tool for Pre and Post Intervention Designs (Brown et al 2014)**

| **Criterion** | **Jacobs et al 2014** | **Lang et al 2016*** | **Roussy et al 2015** |
| --- | --- | --- | --- |
| 1. **Sampling (2 points)** | | | |
| 1. Was probability sampling used? (1 point) | Yes | Yes | No |
| 1. Was sample size justified to obtain power? (1 point) | N/A | No | N/A |
| 1. **Design (2 points)** | | | |
| 1. One pre-test or baseline and several post-test measures (2 points) **OR** | - | - | Yes |
| 1. Simple before-and-after study (1 point) | Yes | Yes |  |
| 1. **Control of confounders (2 points)** | | | |
| Does the study employ a comparison strategy? An attempt to create or assess equivalence of groups at baseline by: |  |  |  |
| 1. Matching group participants (2 points) **OR** | Yes | - | Yes |
| 1. Statistical control (1 point) **OR** | - | Yes | - |
| 1. None (0 point) | - | - | - |
| 1. **Data collection and Outcome measurement (5 points)** | | | |
| 1. Was the dependent variable directly measured by an assessor? (1 point) | No | No | No |
| 1. Were dependent variables either: 2. Directly measured (2 points) **OR** 3. Self-reported (1 point) | Yes (self-report) | Yes (self-report) | Yes (self-report) |
| 1. Were dependent variables measured reliably (with reliability indices previously or for this study)? (1 point) | Yes | Yes | Yes |
| 1. Were dependent variables measured validly (with validity assessments previously or for this study)? (1 point) | Yes | No | No |
| 1. **Statistical analysis and Conclusions (4 points)** | | | |
| 1. Was (were) the statistical test(s) used appropriate for the main outcome and at least 80% of the others (1 point) | Yes | Yes | Yes |
| 1. Were p values and confidence intervals reported properly? (1 point) | Yes | Yes | Yes |
| 1. If multiple outcomes were studied, were correlations analyzed? (1 point) | No | Yes | Yes |
| 1. Were missing data managed appropriately? (1 point) | Yes | N/A | Yes |
| 1. **Drop outs (1 point)** | | | |
| 1. Is attrition rate <30% (if no attrition code1) (1 point) | Yes | N/A | No |
| **Total Score** | **0.73** | **0.57** | **0.67** |
| **Rank** | **Moderate** | **Weak** | **Moderate** |

**Note: Total score calculation (total number of points obtained ÷ 16 – N/A). N/A = not applicable. Highest possible number of points that can be obtained is 16. Articles and papers with a score of 0.80 or higher were rated strong, a score between 0.61-0.79 as moderate, and a score of 0.60 or less as weak. *Included as it was the only paper that reported outcomes at the systems level.**

**Mixed Methods Appraisal Tool (Pluye et al 2011)**

| **Criterion** | **Bazyk et al 2015** | **Keogh et al 2006** | **Mathews & Lynch 2007** | **Ruiz et al 2012** | **Swanson et al 2011** |
| --- | --- | --- | --- | --- | --- |
| **Screening questions (for all types) (no points)** | | | | | |
| 1. Are there clear qualitative and quantitative research questions (or objectives), or a clear mixed methods question (or objective)? | Yes | Yes | Yes | Yes | Yes |
| 1. Do the collected data allow address the research question (objective)? | Yes | Yes | Yes | Yes | Yes |
| **Qualitative (4 points)** | | | | | |
| 1. Are the sources of qualitative data (archives, documents, informants, observations) relevant to address the research question (objective)? | Yes | Yes | Yes | Yes | Yes |
| 1. Is the process for analyzing qualitative data relevant to address the research question (objective)? | Yes | Yes | Yes | Yes | Yes |
| 1. Is appropriate consideration given to how findings relate to the context, e.g., the setting, in which the data were collected? | Yes | Yes | Yes | Yes | Yes |
| 1. Is appropriate consideration given to how findings relate to researchers’ influence, e.g., through their interactions with participants? | No | No | CT | No | No |
| **Quantitative descriptive (4 points)** | | | | | |
| 1. Is the sampling strategy relevant to address the quantitative research question (quantitative aspect of the mixed methods question)? | Yes | Yes | Yes | Yes | Yes |
| 1. Is the sample representative of the population understudy? | Yes | Yes | CT | Yes | Yes |
| 1. Are measurements appropriate (clear origin, or validity known, or standard instrument)? | Yes | Yes | Yes | Yes | Yes |
| 1. Is there an acceptable response rate (60% or above)? | Yes | No | Yes | Yes | Yes |
| **Mixed Methods (3 points)** | | | | | |
| 1. Is the mixed methods research design relevant to address the qualitative and quantitative research questions (or objectives), or the qualitative and quantitative aspects of the mixed methods question (or objective)? | Yes | Yes | Yes | Yes | Yes |
| 1. Is the integration of qualitative and quantitative data (or results) relevant to address the research question (objective)? | Yes | Yes | Yes | Yes | Yes |
| 1. Is appropriate consideration given to the limitations associated with this integration, e.g., the divergence of qualitative and quantitative data (or results) in a triangulation design? | No | No | No | No | No |
| **Total Score** | **75% (0.75)** | **75% (0.75)** | **75% (0.75)** | **75% (0.75)** | **75% (0.75)** |
| **Rank** | **Moderate** | **Moderate** | **Moderate** | **Moderate** | **Moderate** |

**Note: Total score calculation: 25% (QUAL=1 or QUAN=1 or MM=0), 50% (QUAL=2 or QUAN=2 or MM=1), 75% (QUAL=3 or QUAN=3 or MM=2), 100% (QUAL=4 or QUAN=4 or MM=3). CT = can’t tell; QUAL = qualitative component; QUAN = quantitative component; MM = mixed methods component. Articles with a score of 100% (1.00) were rated strong, a score of 75% to 99% (0.99-0.75) as moderate, and a score of 74% (0.74) or less as weak.**
