# Supplementary material for: Effectiveness of capacity building interventions relevant to public health practice: a systematic review
Source: BMC Public Health. 2018 Jun 1;18:684. doi: 10.1186/s12889-018-5591-6 (PMC5984748; doi:10.1186/s12889-018-5591-6)
Supplement: Supplementary file 3 — Table S1. (Characteristics of included papers) to be linked approximately here (DOCX 37 kb) [file 12889_2018_5591_MOESM3_ESM.docx]

| **Author** | **Year** | **Design** | **Population and**  **Setting** | **Intervention** | **Provider(s)** | **Context** | **Theories and Frameworks Cited** | **Theories and Frameworks Applied** |
| --- | --- | --- | --- | --- | --- | --- | --- | --- |
| Bazyk  et al | 2015 | Mixed methods | Occupational therapists (OT) working with children and youth who are part of communities of practice and who represent different practice settings and geographic settings  (Occupational therapists) | 6 month community of practice (CoP) involving a relationship between researchers and practitioners, active learning strategies (reading, face to face, and online discussions), and CoP for community, shared learning and problem solving as well as resource sharing and new strategies for doing.  Three face to face meetings (start, middle, end) in a central location, and six online discussions (reading, reflection, sharing) | Lead OT change leader and three co-facilitators | Individual capacity building  (across a distributed network of OT staff) | WHO public health approach to mental health  CIHR Integrated KT  Communities of Practice | Communities of Practice implemented as a strategy  KT elements applied in strategies used for the intervention |
| Brady & Keogh | 2015 | Qualitative (participatory design) | Community health (community health workers (CHW)) | Pilot education program (i.e., four 3 hour sessions delivered over 4 weeks)  Multi-component (i.e., tutorials, demonstrations, videos, case analysis, small group discussions, role play) | Asthma Society of Ireland and Pavee Point Traveller and Roma Centre | Individual capacity building  (staff working with hard-to-reach populations) | N/A | N/A |
| Cook  et al | 2008 | Meta-analysis | Health professions learners (residents, doctors, nurses, dentist, pharmacists, other) | Internet-based instruction | Not specified | Individual capacity building using Internet-based learning | N/A | N/A |
| Jacobs  et al | 2014 | Pre-post quasi-experimental with comparison group | Local health departments  (health department practitioners) | Evidence based public health training course (modified for local context) and additional capacity building activity (e.g., technical assistance, grant proposal development, program implementation, or evaluation)  Training (in person or via interactive webinars) | Prevention Research Centers and Public Health Practice Based Research Networks or Public Health Training Centers | Individual capacity building | N/A | N/A |
| Kegler & Redmon | 2006 | Qualitative (post-test evaluation) | Various organizations (e.g., CBOs, national organizations, etc.) | Technical assistance (e.g., strategic planning assistance, workshops, conferences, trainings, sustainability planning, needs assessment and evaluation, etc.) | Expert consultants | Individual and organizational capacity building | N/A | N/A |
| Keogh  et al | 2016 | Mixed methods | Health, education and community settings (staff members) | Foundation Programme in Sexual Health Promotion (five 2-day sessions) | Sexual health promotion staff (Irish Health Service Executive) | Individual and organizational capacity building | Life-course approach | Life-course approach applied in intervention content and evaluation |
| Lambraki et al | 2015 | Qualitative | Public health unit  (staff members) | Technical assistance | Ontario health promotion resource centre | Individual capacity building | N/A | N/A |
| Lang  et al | 2016 | Pre-post evaluation | Department of Children and Families  (staff members) | Connecticut Collaborative on Effective Practices for Trauma (CONCEPT):  Child welfare workforce development  Trauma Screening  Trauma informed policy and practice guide revisions  Evidence based practices dissemination | Multidisciplinary core team (i.e., managers, project coordinator, administrators, family partner, evaluation team leads, child trauma implementation leads) | Systems level capacity building | N/A | N/A |
| Mathews & Lynch | 2007 | Mixed methods | Department of Health and Community Services  (public health nurses, social workers, dieticians, rehab therapist, senior managers, psychologists, mental health/addiction counsellors) | Research skills training program (five 1-day workshops held over one year)  Mentoring available during workshop and in-between workshops | First author of article (holds faculty position at Memorial University, Newfoundland) | Organizational research capacity building | N/A | N/A |
| Murad  et al | 2010 | Meta-analysis | Doctors, nurses, residents, other health professions | Educational interventions or curricular interventions using self-directed procedures | Not specified | Individual capacity building using self-directed learning (SDL) methods | N/A | N/A |
| Preskill & Boyle | 2008 | Mixed methods**  (qualitative) | Health and/or human services sector (public health service/  organizations, cancer prevention, county mental health, CBOs)  Education sector (i.e., non-profit or faith-based organizations) | Multi-component (i.e., involvement in an evaluation process; training; technical assistance; written materials; community of practice; mentoring/coaching; internship; appreciative inquiry; use of technology; meetings) | Evaluators and clients (manager or leader) | Organizational evaluation capacity building | N/A | N/A |
| Roussy et al | 2015 | Pre-post with control group | Community health (community health services managers, team leads, and staff members) | **Training (intervention & control)**  Training session (in-person, 3.5 hr.) using case scenarios and individual reflection techniques  **Intervention (intervention only)**  Training session (in-person, 3 hr.) developed and delivered by consumers with dual diagnosis | Senior dual-diagnosis clinician (training session)    Group of 7 individuals living with mental health and substance use issues (intervention session) | Individual capacity building | N/A | N/A |
| Ruiz  et al | 2012 | Mixed methods | Community academic partnership  (community health workers) | Two part 105 hour training program (9 core competencies and specialization training modules)  **Part 1:** 70 hour training held 2 days per week, 8 hours a day over 7 weeks.  **Part 2:** 35 hour specialization training offered as 13 supplemental sessions varying 1.5 to 4 hours; held 1 or 2 times a weeks, over 2 months | Part 1: CHW Network’s executive director, a CHW, and experts with social work experience    Part 2: Academic institution representatives and experienced community health center staff | Individual capacity building | Adult leaning principles  Popular education (as a learning model) | Adult leaning principles applied in curriculum  Popular education applied in use of techniques wherein participants were both teachers and learners, and could learn by experience from the learners’ perspective |
| Swanson et al | 2011 | Mixed methods | National Health Services- Diabetes care  (consultant diabetologists, general practitioners, dietitians, podiatrists, diabetes specialist nurses and primary care nurses) | Workshops (three 1 day sessions) with assigned homework between (2 to 3 weeks) each session | Multidisciplinary team (psychologists diabetologists, specialist general practitioners) with 3 facilitators per session | Individual capacity building | Cognitive behavioural frameworks  Process models of behaviour change | Cognitive behavioural frameworks used in determining content and delivery.  Process models of behaviour change used in setting intervention strategies. |

****Study authors identify methodology as mix methods; article reports findings from a larger study presenting only qualitative data**
