# Supplementary material for: Effectiveness of capacity building interventions relevant to public health practice: a systematic review
Source: BMC Public Health. 2018 Jun 1;18:684. doi: 10.1186/s12889-018-5591-6 (PMC5984748; doi:10.1186/s12889-018-5591-6)
Supplement: Supplementary file 4 — Table S2. (Intervention types and outcomes) to be linked approximately here (DOCX 51 kb) [file 12889_2018_5591_MOESM4_ESM.docx]

| **Intervention type** | **Number** | **Outcomes** | **Findings/Results** |
| --- | --- | --- | --- |
| Training programs/workshops  (Brady 2015, Jacobs 2014, Keogh 2016, Mathews 2007, Roussy 2015, Ruiz 2012, Swanson 2011) | 7 | - Knowledge - Understanding - Skill - Confidence (self-efficacy) - Changes in practice and policies - Behavior change - Application | **Knowledge**   - Agreement among participants in improved knowledge: greater understanding of the causes of asthma and asthma attacks; speaking of inhalers in an informed manner; increased knowledge of accessories for inhalers   Knowledge (N=14 pre-test; 15 post-test)   - No increase in participants’ knowledge scores (pre-test: M=9.3, SD=2.6; post-test M=9.3, SD=3.0, p=0.986 - reports of increased capacity of organization to conduct research   Knowledge and confidence (N=97)   - knowledge of issues of sexual health (M=8.2, SD=1.6, N=97)   **Qualitative Knowledge and confidence (N=22)**   - increase in knowledge and comfort of area   **Qualitative Knowledge and skill development**   - new knowledge and skills/techniques were acquired by many participants (e.g., questioning and listening skills, putting structure around consultations)   **Understanding**   - At baseline there was a significant different between the intervention and control (P<0.05) - After the first training, increase in understanding was seen in the control group; differences in means between the intervention and control was non-significant (p=0.18). - After the second training, there was a 15% significant increase in scores in the intervention group (p<0.01); differences in means between the intervention and control was significant (p<0.01). - Scores remained high at the intervention site 5 months post intervention   **Intervention site (N=23)**   - Baseline (M=10.48, SD =3.35) - Clinician-led training (M=10.52, SD =2.66) - Consumer led training (M=12.00, SD =2.30) - 5 month follow-up (M=12.00, SD =2.05)   **Control site (N=18)**   - Baseline (M=8.28, SD =2.35) - Clinician-led training (M=9.44, SD =2.31) - Consumer led training (M=9.56, SD =2.15) - 5 month follow-up – Analysis not completed due to insufficient numbers   **Skill**  Skills (N=97)   - ability to develop policies around sexual health (M=6.6, SD=2.1, N=92) - problem solving skills around sexual health issues (M=7.4, SD=1.8, N=97) - facilitation skills around sexual health (M=7.9, SD=2.0, N=96) - sexual health promotion skills (M=8.1, SD=1.4, N=97) - ability to communicate about issues of sexual health (M=8.3, SD=1.9, N=97)   Skills (N=14 pre-test; 15 post-test)   - Significant (P>0.05): increases for writing a research question, critically reading research literature, developing a research study, and developing a data-collection tool - Writing a research question (pre-test: M=2.5, SD=1.2; post-test M=3.9, SD=0.8, p=0.003) - Doing literature searches (pre-test: M=2.5, SD=1.2; post-test M=3.9, SD=1.1, p=0.067) - Critically reading research literature (pre-test: M=2.9, SD=1.1; post-test M=3.7, SD=0.8, p=0.047) - Developing a research study (pre-test: M=2.5, SD=1.1; post-test M=3.5, SD=0.7, p=0.008 - Developing a data-collection tool (pre-test: M=2.5, SD=1.4; post-test M=3.9, SD=0.8, p=0.033) - Analyzing data (pre-test: M=3.1, SD=1.2; post-test M=3.8, SD=1.0, p=0.854) - Drawing conclusions from data(pre-test: M=3.2, SD=1.1; post-test M=3.8, SD=0.7, p=0.120) - Writing a research report (pre-test: M=2.8, SD=1.2; post-test M=3.9, SD=0.7, p=0.015) - Presenting findings to others (pre-test: M=3.3, SD=1.3; post-test M=3.9, SD=0.8, p=0.176) - Skills gained applied to other parts of job (e.g., reading critically, incorporating evaluation into programs)   Skill   - baseline(N=81) score for positive communication/behaviour change techniques (M=108.40 [out of 160], SD =16.23) increased at post-intervention(N=59) (M=124.15, SD =10.91; t= 7.17, df=53, p=0.0001) - baseline score (N=81) for negative communication/behaviour change techniques (M=40.54 [out of 80], SD =8.50) reduced at post-intervention (N=59) (M=29.42, SD =8.74; t= 11.13, df=55, p=0.0001)   **Confidence (self-efficacy)**  Knowledge and confidence (N=97)   - confidence in relation to issues of sexual health (M=8.4, SD=1.5,N=97) - motivation to engage with sexual health promotion activities (M=8.5, SD=1.3, N=96)   **Quantitative (n = 12)**   - A 23% improvement in confidence from baseline to post-training across all competencies   **Qualitative (n= 12)**   - themes identified: confidence in ability to utilize skills; intentions related to application of learning (intention to apply learning and intention to change approach in work)   **Qualitative Knowledge and confidence (N=22)**   - reports of increased confidence and motivation of including sexual health promotion activities within their roles - increase in knowledge and comfort of area   **Qualitative Self-development (self-efficacy)**   - increased confidence of skill use and feeling empowered and motivated   **Changes in practice and policies**  Changes in practice (N=43)  **Individual capacity building practices**   - Attending training or education on sexual health promotion (CPD) (before=10 (23%), after=23 (53%), difference=+13 (30%)) - Providing formal sexual health education to clients or service users in a group setting before=14 (33%), after=24 (56%), difference=+10 (23%)) - Referring people to sexual health services (before=24 (56%), after=33 (77%), difference=+9 (19%)) - Assessing sexual health promotion needs of clients (before=26 (60%), after=33 (77%), difference=+7 (17%)) - Providing sexual health education to clients or service users in a one-to-one setting (before=27 (63%), after=33 (77%), difference=+7 (14%))   **Organizational capacity building practices**   - Raising awareness of sexual health promotion needs within your organisation (before=14 (33%), after=32 (74%), difference=+18 (41%)) - Providing formal sexual health education to staff within your organisation (before=1 (2%), after=14 (33%), difference=+13 (31%)) - Developing written information materials related to sexual health (before=4 (9%), after=16 (37%), difference=+12 (28%)) - Developing sexual health services within your organisation (before=6 (14%), after=17 (40%), difference=+11 (26%)) - Auditing sexual health promotion practices within your organisation (before=4 (9%), after=12 (28%), difference=+8 (18%)) - -Assessing sexual health promotion education needs of staff within your organisation (before=5 (12%), after=13 (30%), difference=+8 (18%)) - -Developing sexual health policies/guidelines within your organisation (before=5 (12%), after=12 (28%), difference=+7 (17%)) - Engaging in research on sexual health (before=5 (12%), after=13 (30%), difference=+8 (18%)) - Adapting administrative practice to include sexual health items, for example, developing/changing assessment forms to include sexual health (before=2 (5%), after=7 (16%), difference=+5 (11%))   **Inter-organizational capacity building practices**   - -Networking about sexual health with other individuals or organisations (before=10 (23%), after=27 (63%), difference=+17 (40%)) - -Publishing articles on sexual health (before=2 (5%), after=7 (16%), difference=+5 (11%)) - -Developing sexual health policies/guidelines outside of your organisation (before=0 (0%), after=3 (7%), difference=+3 (7%)) - -Providing formal sexual health education to staff outside your organisation (before=2 (5%), after=5 (12%), difference=+3 (7%)) - -Developing sexual health services outside your organisation (before=1 (2%), after=3 (7%), difference=+2 (5%)) - -Average number of activities reported per person (mean/SD) and difference before/after (before=3.77 (2.60), after=7.60(3.99), difference=+3.83   **Qualitative Change in practice (N=22)**  **Organizational and inter-organizational capacity building practices**   - organizational capacity building activities mainly focused on educating other staff and increasing awareness of sexual health issues (e.g., conducing workshops and trainings for staff; use of facilitation skills; staff discussion on sexual health concepts) - some reports of policy and guideline development - a few reports of inter-organizational activities (e.g., journal publications; workshops for other organizations, training for volunteers; formal and informal networking; conducting research)   **Behavior change**  **Qualitative Self-development (self-efficacy)**   - implementation of changes in behaviour as well as giving some thought to change for the future and “food for thought”   **Qualitative Knowledge and skill development**   - some reports of changes in behavior   **Application**   - Participants applied knowledge learned: educated their family members about correctly managing asthma and proper use of inhalers; educating community members - Participants educated other community health workers (in Traveller community) using a train the trainer model   **On average, every month since the EBPH course I have (N = 98):**   - -Searched the scientific literature for information on programs (35.7%) - -Used the EBPH materials/skills in planning a new program (26.5%) - -Used the EBPH materials/skills in modifying an existing program (24.5%) - -Used the EBPH materials/skills in evaluating a program (23.5%) - -Referred to the EBPH readings that were provided (22.4%) - -Used the EBPH materials/skills for grant applications (3.1%)   **The EBPH course content helped me (N=98):**   - -See applications for this knowledge in my work (92.9%) - -Become a better leader who promotes evidence-based decision making (86.7%) - -Acquire knowledge about a new subject (85.7%) - -Make scientifically informed decisions at work (80.6%) - -Communicate better with co-workers (65.3%) - -Read reports and articles (63.3%) - -Adapt an intervention to a community's needs while keeping it evidence based (63.3%) - -Develop a rationale for a policy change (62.2%) - -Teach others how to use/apply the information in the --EBPH course (61.2%) - -Identify and compare the costs and benefits of a program or policy (60.2%) - -Implement evidence-based practices in CDC cooperative agreement or other funded programs (51.0%) - -Obtain funding for programs at work (39.8%) |
| Multi-strategy interventions    (Lang 2016, Preskil 2008 ) | 2 | - Knowledge - Skill - Perception of system level capacity | **Knowledge**   - Majority reported of general increase in knowledge and skills of evaluation (e.g., developing logic models; understanding of evaluation terms and concepts and importance of findings for decision making; different types of data collection methods; understanding of evaluation process)   **Skill**   - All participants identified that some type of skill was developed. - Most reported that staff ask better questions (more frequently too) about programs; as well as more use of evaluation findings - Some reports of better ability of staff to design data collection tools, and more efficient communication and reporting with stakeholder   **Perception of system level capacity**   - Mean scores for majority of domains had a significant increase from year 1 to year 3 - Trauma training and education (Mean 0. 39, 95% CI -0.23 to 0.54, p<0.01) - Staff trauma knowledge and practice Mean 0. 26, 95% CI 0.11 to 0.40, p<0.01) - Individual trauma knowledge and practice (Mean 0. 33, 95% CI 0.20 to 0.46, p<0.01) - Trauma supervision and supports (Mean 0. 52, 95% CI 0.33 to 0.0.71, p<0.01) - Staff supports child relationships (Mean 0. 24, 95% CI 0.09 to 0.41, p<0.01) - Birth family trauma support (Mean 0. 46, 95% CI 0.31 to 0.61, p<0.01) - Resource family trauma support (Mean 0. 42, 95% CI 0.28 to 0.56, p<0.01) - Staff addresses child psychological safety (Mean 0.18, 95% CI 0.01 to 0.34, p<0.05) - Agency trauma assessment (Mean 0. 39, 95% CI 0.20 to 0.58, p<0.01) - Access to trauma-informed services (Mean 0. 51, 95% CI 0.33 to 0.69, p<0.01) - Local agency collaboration (general) (Mean 0. 05, 95% CI -0.06 to 0.16, **non-significant**) - Local agency collaboration (trauma) (Mean 0. 31, 95% CI 0.15 to 0.48, p<0.01) |
| Technical assistance  (Kegler 2006, Lambraki 2015) | 2 | - Knowledge - Skill - Leadership (self-efficacy) - Confidence (self-efficacy) - Changes in practice and policies - Organizational support | **Knowledge and skill**   - **Prominent theme:** Increased knowledge on topics related to basics of tobacco control and prevention - **Other themes:** Improved communication and presentation skills - Reported gains in knowledge relevant to work - Gained familiarity with acronyms used in tobacco control - Development of new skills (e.g., overcoming barriers at council meetings; writing annual plans using SMART objectives; skills in media and communication such as developing tobacco-related communication plans) - Incorporating and using evidence in work (e.g., proposals, presentations   **Leadership (self-efficacy)**   - Reports of strengthened leadership abilities through improved knowledge, skill or understanding of tobacco control - Improved confidence in work and improvement in cultural competence   **Confidence (self-efficacy)**   - increased confidence in work (through feedback by TA providers) - commitment to take on new challenges and overcome barriers   **Changes in practice and policies**   - Services improved or changed tobacco control practices through strategic planning practices (e.g., greater strategic focus, development of partnerships and expanded networks) - Mixed reviews on increased support from policy makers or reports of no increase - Services helped with foundational work for policy action   **Organizational support**   - Neutral and mixed-feelings about impact of services on organizational support |
| Community of Practice  (Bazyk 2015) | 1 | - Knowledge - Changes in practice and policies | **Knowledge**  Quantitative: 117/185 completed post survey  Knowledge statements: significant change (p<0.00)  Belief statements: significant change (p<0.00 or p<0.02)  Action statements: significant change pre to post test (p<0.00)  **Changes in practice and policies**   - Capacity building was meaningful and enjoyable (described changes to daily practice; perception of support via networks) - Reported change in thinking because of new knowledge wrt mental health (“reframing”, “paradigm shift”, plans to use new knowledge - Experience evoked strong emotions re: OT practitioners’ identities (reconnection to mental health roots was rewarding, enhanced awareness of scope of practice of OT) - Reported changes in practice (ways of working shifted, including large changes to their own work such as undertaking a new, year-long program, joining new initiatives) |
| Educational/curricular interventions using SDL  (Murad 2010) | 1 | - Knowledge - Skill | **SDL vs. traditional teaching methods**  **Knowledge**  **All participants including medical students:**  Moderate increase in knowledge (SMD 0.45, 95% CI 0.23 to 0. 67)  **Sub-group analysis:**  Doctors (SMD 0.50, 95% CI -0.16 to 1.17)  Nurses (SMD 1.60, 95% CI 0.69 to 2.52 )  Residents (SMD 0.49, 95% CI -0.28 to 1.25)  Other health professionals (SMD 0.16, 95% CI -0.34 to 0. 66)  P = 0.11  **Skill**  **All participants including medical students:**  Non-statically significant increase in skills (SMD 0.05, 95% CI 0.05 to 0. 22)  **Sub-group analysis:**  Doctors (SMD 0. 60, 95% CI -0.47 to 0.59, N=1)  Residents (SMD 0.16, 95% CI -0.60 to 0.92, N=1)  Other health professionals (SMD -0.14, 95% CI -0.64 to 0. 37, N=2)  P = 0.84 |
| Internet-based instruction  (Cook 2008) | 1 | - Knowledge - Skill | **Internet-based learning vs. no interventions**  **Knowledge**  **All participants including students:**  Benefit on learners knowledge (ES 1.00, 95% CI 0.90 to 1.10, p<0.001 N=127)  **Sub-group analysis:**  Doctors (ES 1.02, 95% CI 0.88 to 1.15 N=58)  Nurses (ES 0.88, 95% CI 0.69 to 1.06 N=21)  Other (ES 1.20, 95% CI 0.98 to 1.43 N=32)  **Skill**  **All participants including students:**  Skills (ES 0.85, 95% CI 0.49 to 1.20, p<0.001 N=16)  Interventions with practice exercises higher than those without (ES 1.01, 95% CI 0.60 to 1.43 vs. ES 0.21, 95% CI 0.04 to 0.38 vs. p<0.001)  **Sub-group analysis:**  Doctors (ES 1.19, 95% CI 0.19 to 2.19 N=3)  Nurses (ES 0.72, 95% CI -0.03 to 1.47 N=3)  Other (ES 0.87, 95% CI 0.43 to 1.30 N=6)  **Internet-based learning vs. non-internet interventions**  **Knowledge**  **All participants including students:**  (ES 0.12, 95% CI 0. 003 to 0.24, p=0.045 N=36)  Sensitivity analysis excluding blended interventions  (ES 0.065, 95% CI -0. 062 to 0.19, P = 0.31)  **Sub-group analysis:**  Doctors (ES 0.12, 95% CI -0.10 to 0.33 N=9)  Nurses (ES 0.09, 95% CI -0.13 to 0.32 N=17)  Other (ES 0.01, 95% CI -0.17 to 0.18 N=26)  **Skill**  **All participants including students:**  Skills (ES 0.09, 95% CI -0.26 to 0.44, p=0.61 N=12)  Interventions with practice exercises higher than those without (ES 1.01, 95% CI 0.60 to 1.43 vs. ES 0.21, 95% CI 0.04 to 0.38 vs. p<0.001)  **Sub-group analysis:**  Doctors (ES 0.44, 95% CI -0.01 to 0.89 N=2)  Nurses (ES 0.03, 95% CI -0.59 to 0.64 N=6)  Other (ES 0.02, 95% CI -0.47 to 0.51 N=4) |
